# Osteomyoplastic transtibial amputation: technique and tips

**DOI:** 10.1186/1749-799X-6-13

**Published:** 2011-03-07

**Authors:** Benjamin C Taylor, Attila Poka

**Affiliations:** 1 Department of Orthopaedic Surgery, Grant Medical Center, 285 East State Street, Suite 500, Columbus, OH, 43215, USA

## Abstract

Treatment of severe lower extremity trauma, diabetic complications, infections, dysvascular limbs, neoplasia, developmental pathology, or other conditions often involves amputation of the involved extremity. However, techniques of lower extremity amputation have largely remained stagnant over decades.

This article reports a reproducible technique for transtibial osteomyoplastic amputation.

## Background

Amputation osteomyoplasty, or bone bridging, is a technique developed in 1920 to better correct the residual limb to a normal physiological status [1]. Proponents of this technique state that the bone bridging between the tibia and fibula creates a larger and more stable end-bearing construct as well as preventing the fibular instability that occurs secondary to loss of the ankle mortise [2-7]. Vascularity of the residual limb is improved by sealing the intramedullary canal, which has been shown in angiographic studies to reestablish intramedullary pressure, improve medullary blood flow comparable to healthy volunteers and increase the blood flow to the residual limb [3,8-10]. The myoplasty or myodesis component of the procedure recreates the normal length-tension of the muscles [2,4,7], increases and stabilizes the surface area available for prosthetic fitting[11], normalizes muscle function as viewed with EMG testing [12], and improves both the arterial and venous circulation of the residual stump [8,13,14].

## Results

The patient is placed in the supine position and a general anesthetic administered. A pneumatic tourniquet is placed on the proximal thigh and a bump under the ipsilateral buttock is helpful to control rotation of the limb.

Incision site and flap creation will depend on location of scars, deformities, wounds, or previous amputations. Approximately twelve to fifteen centimeters of residual tibia should be the goal in an average patient; distal third amputations should be avoided due to poor soft tissue coverage. Seventeen to twenty-two centimeters between the end of limb and the ground is required for the use of most modern integrated high-impact foot and pylon shock-absorbing systems. Preoperative discussion with the patient's prosthetist is recommended to integrate the fitting needs into the surgical plans.

**Figure 1:**
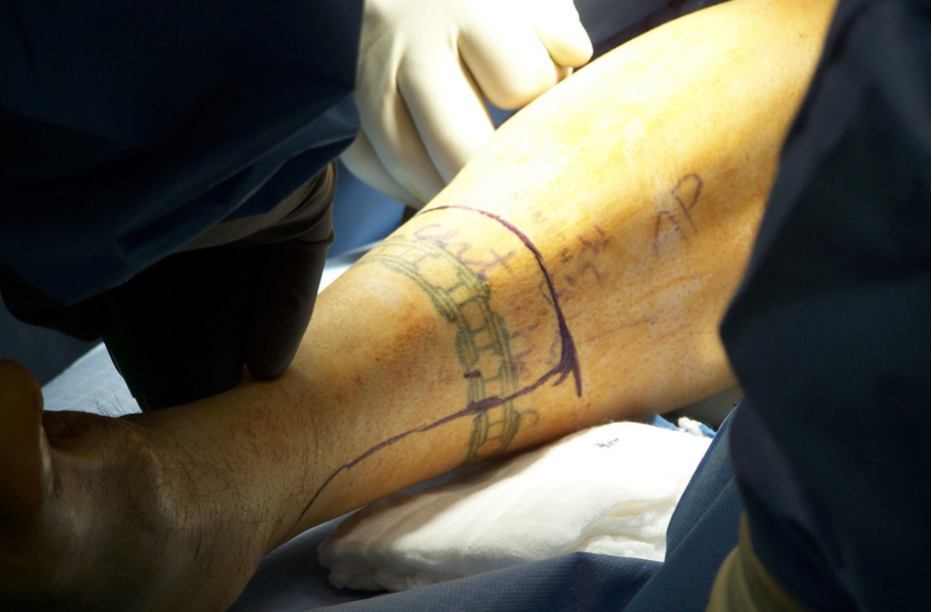
**Skin incision marked to create long posterior flap**.

Although vascular-based skew flaps, fish mouth flaps, long medial flaps or sagittal flaps may be used, we prefer a long posterior flap. For creation of a long posterior flap, the anterior incision is made at the approximate level of resection, whereas the posterior incision is made at a level one to two centimeters distal than the diameter of the leg at the level of bone division (Figure 1). The anterior flap is carried down anteromedially to just above the periosteum as a single layer and the anterolateral muscles are divided down to the intramuscular septum. The anterior tibial vessels and deep peroneal neurovascular structures are individually ligated and divided as they are encountered.

A periosteal flap is created from the anteriomedial and anterolateral surfaces of the tibia from distal to proximal; this is elevated to a level just proximal to the desired tibial cut. If no substantial periosteum is seen, an osteoperiosteal flap can be created with use of an osteotome to lift 1-2 mm of cortical bone on its limited attachment. Proximal attachment of this periosteal flap is desired to ensure maintenance of vascular supply. The tibia is then sectioned with the fibular cut being made approximately three centimeters distal to the level of the tibial cut. The distal tibial piece is then levered anteriorly as the posterior tibia and fibula are released to the level of the posterior flap incision. The nerves and vessels are again individually ligated and divided, and the posterior incision is then carried through in a full-thickness manner.

**Figure 2:**
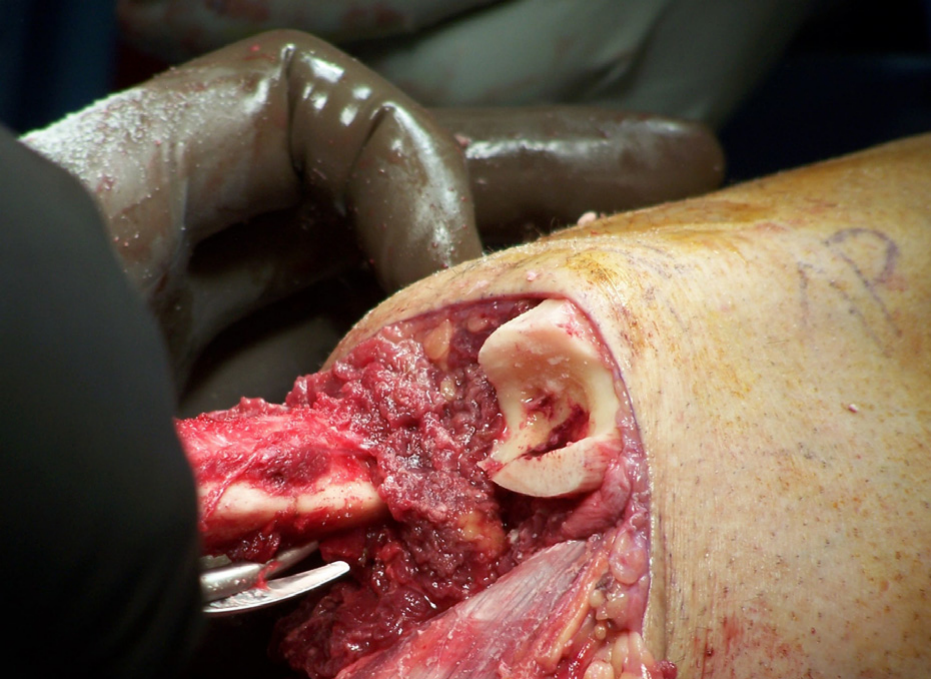
**Provisional notch created in the distal tibia to receive the fibular strut**.

**Figure 3:**
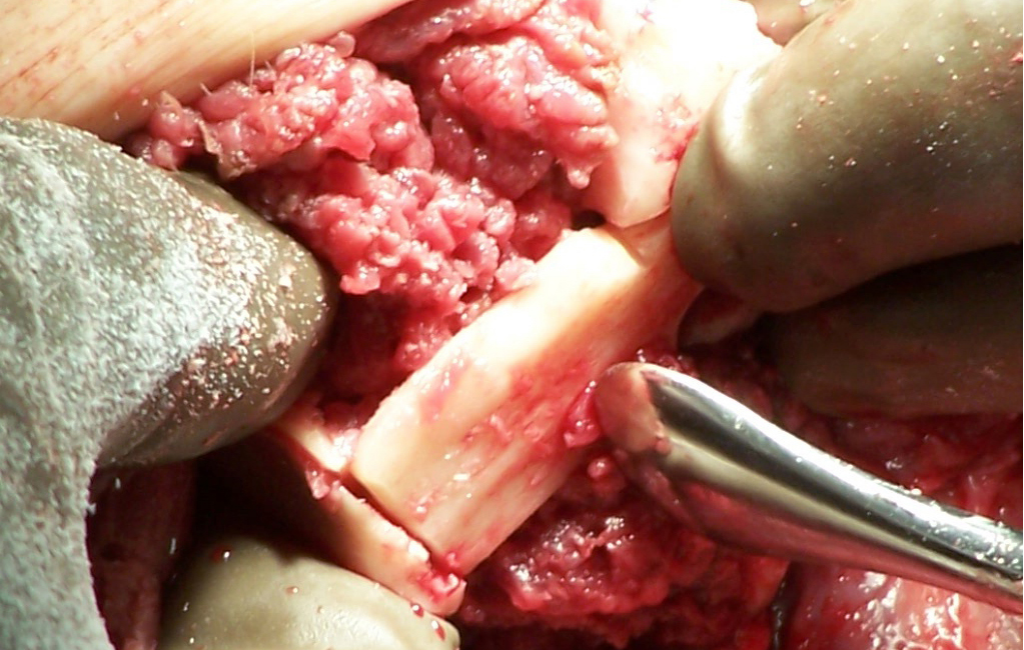
**Fibular strut fitting into the tibial and fibular notches created by the high-speed burr**.

**Figure 4:**
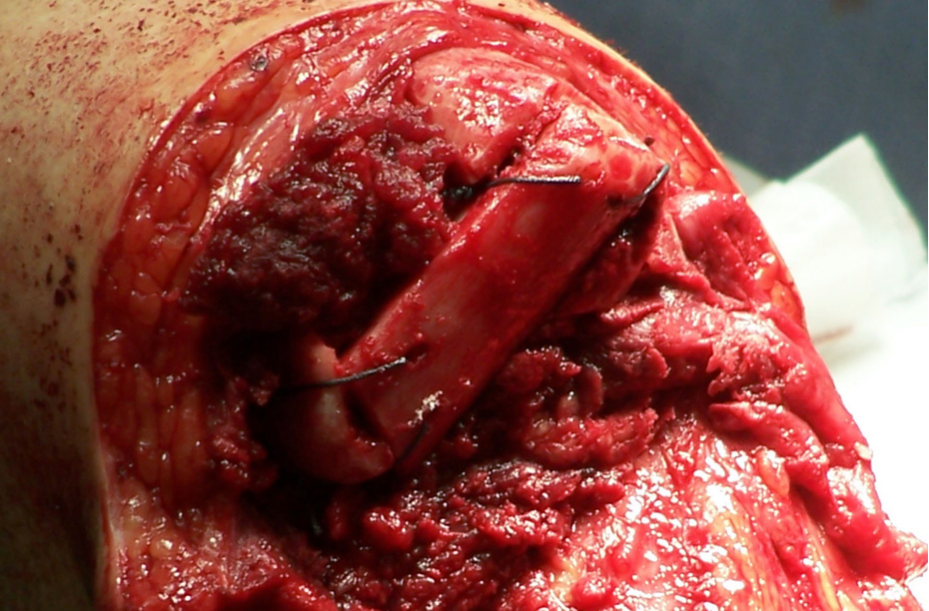
**Fibular strut securely sutured in place via bone tunnels through the fibular strut, distal tibia and fibula**.

A provisional notch to receive the fibula is made in the distal tibia with a high-speed burr (Figure 2). A periosteal flap is then elevated from the remaining fibula and reflected proximally to a level just above the tibial cut. The resting distance between the tibia and fibula at the tibial cut level is then measured (usually between 1-1.5 cm). A second fibular osteotomy is then made; the lateral cortex is osteotomized at the level of the tibial cut with the medial cortex being osteotomized in a step-cut fashion more proximally, to allow an improved fit of the fibular strut. The free fibular piece is then shortened to fit appropriately when laid in a transverse fashion and the tibial groove modified with the high-speed burr as necessary to create a tight fit (Figure 3). The fibular strut is then attached to the fibula and tibia with heavy non-absorbable suture via 2 mm drill holes. A high-speed burr is then used on the distal tibia, fibula and bridge to round and bevel any edges (Figure 4). All periosteal flaps are then carried distally around the bone bridge as a sleeve, and sutured in position.

**Figure 5:**
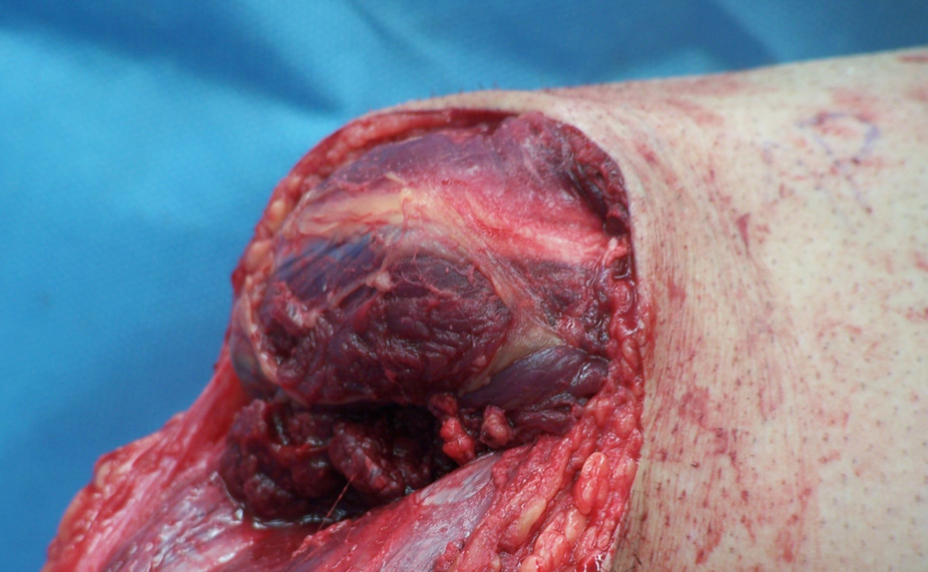
**The peroneal myoplasty is seen in its completed state, with the optimal resting length and tension of the muscles restored**.

**Figure 6:**
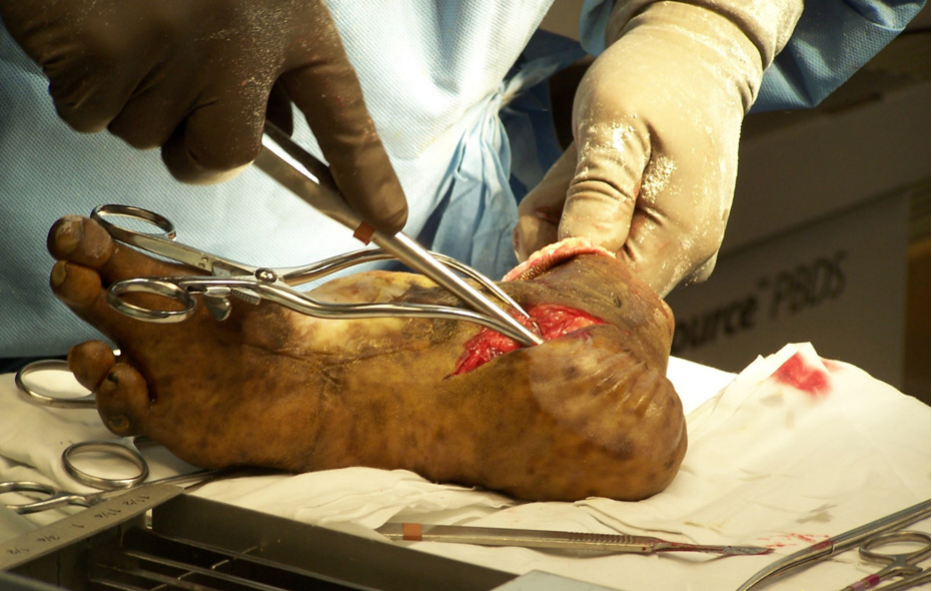
**Harvesting cancellous autograft from the removed aspect of the limb should be considered if the bone is free of infection and graft is needed**.

**Figure 7:**
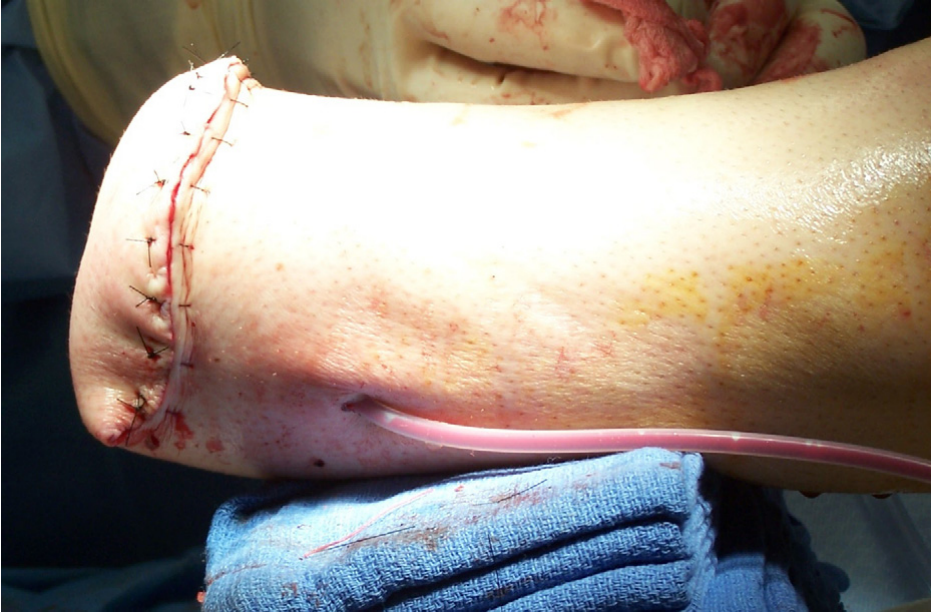
**Final closure without significant tension on wound edges; suction drain also shown in place**.

The tourniquet is released at this time and all bleeding points are clamped and ligated or electrocoagulated appropriately. The peroneal muscles are cut at an appropriate length and brought medially, where they are sutured to the deep fascia and periosteum overlying the anteromedial tibia (Figure 5). Adjunct osteobiological agents may be used in the bony bridge area at this time; the authors have used rhBMP-2, platelet rich plasma, allograft bone, autologous cancellous bone, and combinations thereof in various scenarios. Autograft may also be obtained from the distal stump at this time (Figure 6). A closed suction drain is then placed superficial to the peroneal musculature and carried out of the skin on the anterolateral aspect of the distal stump. The posterior myocutaneous flap is brought anteriorly, evaluated for length and trimmed appropriately. The gastrosoleus muscle complex is then beveled posteriorly as needed, and rotated anteriorly, where it is sutured into the anterior muscle compartment, deep anterior fascia, and periosteum. Skin flaps are fashioned as necessary for a smooth closure without tension and sutured together with interrupted nonabsorbable sutures (Figure 7). Any dog-ears should be trimmed sparingly as to minimize vascular insults to the remaining skin.

## Discussion

The efforts of creating a distal bone bridge and the osteomyoplasty does add time and potential morbidity to the transtibial amputation procedure, but is directed at creating a more functional and physiological residual extremity. Patient reported outcomes from this procedure are encouraging and generally higher than that for traditional transtibial amputees, with improved rate of return to work as well as patient-reported outcomes [1,2,7,15].

Indications for this procedure include acute trauma as well as sequelae from tumor, trauma, previous surgery, and congenital deformities. Although traditional thought is that diabetic or dysvascular patients should not undergo this procedure, several reports of these patients included in larger groups reveal that they can undergo this procedure successfully but may not perform as well on functional testing [1,2,4,6,7].

## Conclusions

The foot is a very unique end-bearing organ, and the removal of the distal limb creates several difficulties. Traditional transtibial amputation creates a smaller and possible less stable area for weightbearing with surrounding soft tissues that are not designed to resist the compressive and shearing forces of weightbearing. This procedure was developed to help create a more enhanced and physiological weightbearing platform.

## Consent

Written informed consent was obtained from the patient for publication of this report and accompanying images. A copy of the written consent is available for review by the Editor-in-Chief of this journal.

## Competing interests

The authors declare that they have no competing interests.

## Authors' contributions

BCT was the primary author of the manuscript. AP contributed to the manuscript and described his technique of amputation. All authors have read and approved the manuscript.
